# Acute Exercise-Induced Response of Monocyte Subtypes in Chronic Heart and Renal Failure

**DOI:** 10.1155/2014/216534

**Published:** 2014-12-22

**Authors:** Amaryllis H. Van Craenenbroeck, Katrijn Van Ackeren, Vicky Y. Hoymans, Johan Roeykens, Gert A. Verpooten, Christiaan J. Vrints, Marie M. Couttenye, Emeline M. Van Craenenbroeck

**Affiliations:** ^1^Laboratory of Cellular and Molecular Cardiology, Antwerp University Hospital, 2650 Antwerp, Belgium; ^2^Department of Nephrology, Antwerp University Hospital, 2650 Antwerp, Belgium; ^3^Laboratory of Experimental Medicine and Pediatrics, University of Antwerp, 2650 Antwerp, Belgium; ^4^Cardiovascular Diseases, Department of Translational Pathophysiological Research, University of Antwerp, 2650 Antwerp, Belgium; ^5^S.P.O.R.T.S., Antwerp University Hospital, 2650 Antwerp, Belgium; ^6^Department of Cardiology, Antwerp University Hospital, 2650 Antwerp, Belgium

## Abstract

*Purpose*. Monocytes (Mon1-2-3) play a substantial role in low-grade inflammation associated with high cardiovascular morbidity and mortality of patients with chronic kidney disease (CKD) and chronic heart failure (CHF). The effect of an acute exercise bout on monocyte subsets in the setting of systemic inflammation is currently unknown. This study aims (1) to evaluate baseline distribution of monocyte subsets in CHF and CKD versus healthy subjects (HS) and (2) to evaluate the effect of an acute exercise bout. Exercise-induced IL-6 and MCP-1 release are related to the Mon1-2-3 response. *Methods*. Twenty CHF patients, 20 CKD patients, and 15 HS were included. Before and after a maximal cardiopulmonary exercise test, monocyte subsets were quantified by flow cytometry: CD14^++^CD16^−^CCR2^+^ (Mon1), CD14^++^CD16^+^CCR2^+^ (Mon2), and CD14^+^CD16^++^CCR2^−^ (Mon3). Serum levels of IL-6 and MCP-1 were determined by ELISA. *Results*. Baseline distribution of Mon1-2-3 was comparable between the 3 groups. Following acute exercise, %Mon2 and %Mon3 increased significantly at the expense of a decrease in %Mon1 in HS and in CKD. This response was significantly attenuated in CHF (*P* < 0.05). In HS only, MCP-1 levels increased following exercise; IL-6 levels were unchanged. Circulatory power was a strong and independent predictor of the changes in Mon1 (*β* = −0.461, *P* < 0.001) and Mon3 (*β* = 0.449, *P* < 0.001); and baseline LVEF of the change in Mon2 (*β* = 0.441, *P* < 0.001). *Conclusion*. The response of monocytes to acute exercise is characterized by an increase in proangiogenic and proinflammatory Mon2 and Mon3 at the expense of phagocytic Mon1. This exercise-induced monocyte subset response is mainly driven by hemodynamic changes and not by preexistent low-grade inflammation.

## 1. Introduction

Monocytes are keystones of the immune system linking innate and adaptive immunity and are critical drivers in inflammatory diseases. Recently, 3 functionally distinct monocyte subsets were identified based on the expression of CD14 (lipopolysaccharide receptor), CD16 (FcyRIII receptor), and CCR2 (receptor for monocyte chemotactic protein-1 (MCP-1)) [1, 2]. Classical monocytes (Mon1, CD14^++^CD16^−^CCR2^+^) are highly phagocytic and are important in first line defense. Intermediate monocytes (Mon2, CD14^++^CD16^+^CCR2^+^) possess proangiogenic properties, have been implicated in antigen processing and presentation, and produce high levels of pro- and anti-inflammatory mediators (TNF*α*, IL-10). Nonclassical monocytes (Mon3, CD14^+^CD16^++^CCR2^−^) have anti-inflammatory effects and play a role in the adaptive immune system as well as in patrolling the endothelial-blood interface [1, 3, 4].

Chronic low-grade inflammation, often objectified by an elevation in circulating levels of proinflammatory cytokines such as interleukin-6 (IL-6) and monocyte chemotactic protein-1 (MCP-1), has been recognized as the underlying factor in the development and progression of various diseases, including cardiovascular disease [5]. Chronic kidney disease (CKD) and chronic heart failure (CHF) share common mechanisms that explain the high cardiovascular morbidity and mortality, including inflammation and oxidative stress-induced vascular dysfunction [6, 7]. Exercise training is a potent strategy for lowering long-term cardiovascular risk and morbidity in sedentary CKD patients [8] as well as mortality in CHF [9]. Randomized controlled exercise-intervention studies have shown that increased physical activity is associated with reduced systemic inflammation in the setting of CHF [10] and CKD [11]. It has been suggested that changes in the proportions of monocyte subsets contribute to this finding.

Although exercise training provides a strong anti-inflammatory effect, each intense exercise bout induces a transient increase in inflammatory markers, such as leukocytosis, monocytosis, and a raise in MCP-1 and IL-6 levels [12–14]. Nevertheless, endurance athletes have lower resting levels of inflammatory markers, including monocytes, in comparison to physically inactive adults, which suggests that the immune system adapts to repetitive exercise bouts [15].

In healthy subjects performing a short strenuous exercise bout, CD16^+^ monocytes are mobilized from the marginal pool within 15 minutes [16]. The effect of acute exercise on the 3 monocytes subsets in CKD and CHF with demonstrated low-grade inflammation is currently unknown. The present study investigates whether monocyte subset distribution and their response to an acute exercise bout are influenced by the presence of chronic low-grade inflammation, the different homeostatic milieu of CKD, or the hemodynamic alterations in CHF. We have the following aims: (1) to explore baseline differences in the monocyte subset distribution between CKD, CHF, and healthy controls; (2) to evaluate the effect of a single maximal exercise bout on the mobilization of Mon1, Mon2, and Mon3 in patients with CKD or CHF in comparison to healthy subjects; and (3) to investigate possible mediators of this response.

## 2. Methods

### 2.1. Subjects

Twenty sedentary CKD patients (estimated glomerular filtration rate (eGFR) < 60 mL/min/1.73 m^2^ or kidney damage for ≥3 months, defined by structural or functional renal abnormalities), 20 sedentary CHF patients (left ventricular ejection fraction (LVEF) ≤ 45%, NYHA classes II-III), and 15 healthy subjects (HS) (no relevant medical history, no pharmacological treatment, and normal ECG and echocardiographic assessment) were enrolled in this study. Patients were stable with regard to symptoms and therapy and were on standard medical treatment.

Exclusion criteria were active inflammatory or malignant disease and treatment with immunosuppressive agents. In the CKD group, the presence of cardiovascular disease, including coronary, peripheral, and cerebrovascular disease, served as an exclusion criterion. Likewise, in the CHF group, patients with impaired renal function (eGFR < 60 mL/min/1.73 m^2^) were excluded.

CHF was due to idiopathic dilated cardiomyopathy in 65% of patients; the remainder was caused by ischemic heart disease. Etiology of CKD included autosomal dominant polycystic kidney disease (25%), the presence of a unique functional kidney (25%), reflux nephropathy (20%), IgA nephropathy (10%), nephroangiosclerosis (10%), focal segmental glomerulosclerosis (5%), and obstructive nephropathy secondary to chronic lithiasis (5%).

### 2.2. Study Design

Subjects were asked to refrain from excessive physical exertion for 24 hours prior to the study. They were called in for a symptom-limited cardiopulmonary exercise test (CPET) on a graded bicycle ergometer. Immediately before and 10 minutes after peak exercise, venous blood samples were drawn from an antecubital vein and collected in EDTA and serum separator tubes. Samples for flow cytometric analysis were processed within 1 hour after collection and serum was stored at −80°C for batch analysis.

All subjects underwent standard transthoracic cardiac ultrasound for the assessment of left ventricular end-diastolic diameter (LVEDD), left ventricular ejection fraction (LVEF), diastolic function (E/é), and right atrial pressure (RAP) using an iE33 echocardiography scanner (Philips Medical Systems, Eindhoven, the Netherlands).

The study was conducted according to the principles outlined in the declaration of Helsinki and was approved by the Ethics Committee of the Antwerp University Hospital. All participants gave written informed consent.

### 2.3. Cardiopulmonary Exercise Testing

Exercise capacity was assessed by a symptom-limited graded exercise test on a bicycle ergometer (CKD and CHF patients: Cardiovit CS-200 Ergo-Spiro, Schiller AG, Baar, Switzerland; healthy subjects: Excalibur Sport ergometer, Lode, Groningen, the Netherlands, and PowerCube-ergo, Ganshorn, Niederlauer, Germany). An individualized ramp protocol, starting with either 20 or 40 Watts and an incremental load of 10 or 20 Watts per minute, was chosen to ensure an optimal duration of the exercise test between 8 and 10 minutes. Twelve-lead ECG was recorded continuously and blood pressure was measured every 2 minutes. Breath-by-breath gas exchange measurements allowed online determination of ventilation (VE), oxygen uptake (VO_2_), and carbon dioxide production (VCO_2_). Peak oxygen consumption (VO_2_peak) was determined as the highest attained VO_2_ during the final 30 seconds of exercise. VO_2_peak and maximal workload were also expressed as a percentage of the predicted value (% predicted VO_2_peak, % predicted Wattmax), according to the nomogram of Hansen et al. [17]. Subjects were encouraged to exercise upon exhaustion, according to the respiratory exchange ratio (RER) and identification of the anaerobic threshold (AT, V-slope method). Circulatory power (VO_2_peak × peak systolic blood pressure) and maximal work-economy (Wattmax/VO_2_peak) were calculated.

### 2.4. Flow Cytometric Quantification of Monocyte Subsets

Monocyte subsets were defined as CD14^++^CD16^−^ CCR2^+^ cells (Mon1), CD14^++^CD16^+^CCR2^+^ (Mon2), and CD14^+^CD16^++^CCR2^−^ (Mon3). Whole blood was incubated with fluorochrome-conjugated antibodies for 15 minutes in the dark. The following antibodies were used: anti-CD14 phycoerythrin (PE), anti-CD16 fluorescein isothiocyanate (FITC), anti-CCR2 allophycocyanin (APC) (R&D Systems, Minnesota, USA), anti-CD45 allophycocyanin-Hilite 7 (APC-H7), and anti-CD86 peridinin chlorophyll protein-cyanine 5.5 (PerCP-Cy5.5). All antibodies were purchased from BD Biosciences (Erembodegem, Belgium) unless stated otherwise. The optimal concentration for each antibody was determined prior to the study by titration assays.

After red blood cells lysis with BD lysing solution (BD Biosciences, Erembodegem, Belgium), the specimen was analysed on a BD FACSCantoII flow cytometer (BD Biosciences, Erembodegem, Belgium). Besides the regular forward scatter (FSC) threshold, an additional threshold was set on APC-H7 (below the CD45 APC-H7 fluorescence of granulocytes) to ensure proper recording of CD45^+^ events in this lyse-no-wash setting. A minimum of 200 000 CD45^+^ events was recorded.

**Figure 1:**
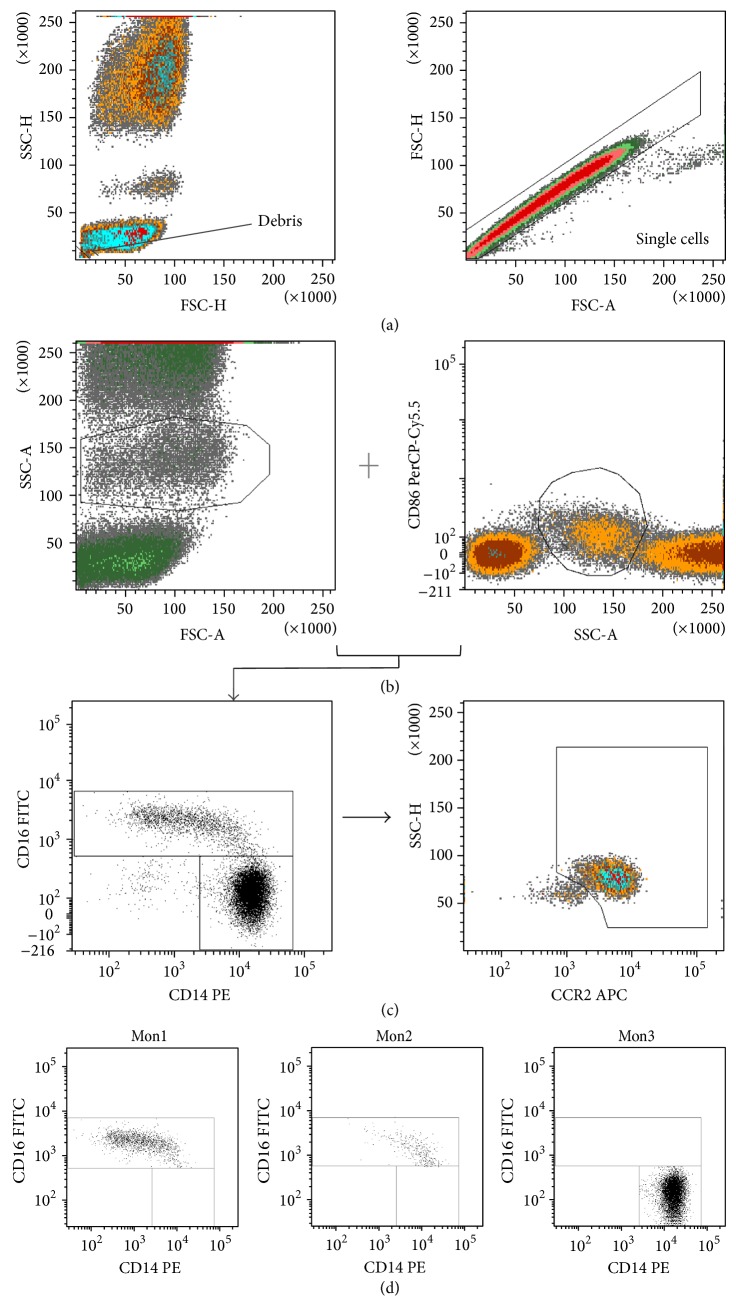
Gating strategy. Gating strategy and presentation of monocyte subsets. (a) Exclusion of debris and doublets. (b) Identification of monocytes based on the forward scatter (FSC) versus side scatter (SSC) plot and CD86 positivity. (c) Separation of monocytes in CD16 positive and CD16 negative monocytes and subsequent distinction between Mon2 and Mon3, based on CCR2 expression. The plot shows all monocyte subsets, with Mon1 as per definition in the CCR2^+^ gate. (d) Respective location of the monocyte subsets on CD14 versus CD16 plot.

Analysis of monocyte subsets was done using BD FACSDiva software version 6.1.2 by a single operator in analogy to the gating strategy of Shantsila et al. [3]. Primary gates were established for monocytes based on expression of the pan-monocytic marker CD86 as well as scatter profile. Secondary gates were established within the monocyte gate to identify CD14^+^ and CD16^+^ cells. CD16^+^ cells were then separated in Mon2 and Mon3 based on their CCR2 expression.  Figure 1 shows a representative example of the applied gating strategy. Total leukocyte count was performed using an automated hematology analyzer (Advia 2120, Bayer HealthCare, Tarrytown, NY). Absolute count of monocytes and their subsets was calculated by multiplying the respective percentages acquired by flow cytometry by the total leukocyte count. Monocyte subsets were expressed as cells/*μ*L and as a percentage of total monocyte count. For lymphocyte and neutrophil percentage, results of the automated hematology analyzer were used.

### 2.5. Biochemical Assays

Creatinine, total cholesterol, high-density lipoprotein (HDL), low-density lipoprotein (LDL), and triglycerides were measured using routine laboratory techniques. Estimated glomerular filtration rate (eGFR) was calculated using the CKD-EPI formula [18].

Serum levels of MCP-1 and IL-6 were analysed in batch by sandwich enzyme-linked immunosorbent assay (Quantikine ELISA kit, R&D Systems, Minnesota, USA). For MCP-1, the intra-assay coefficient of variation (CV) was <10% with a sensitivity of 1.7 pg/mL. For IL-6, the intra-assay CV was <20% with a sensitivity of 0.7 pg/mL.

### 2.6. Statistical Analysis

Continuous data are expressed as mean ± standard deviation (SD). Normality of data was assessed using a one-sample Kolmogorov Smirnov. Baseline characteristics were compared using Chi-square test or one-way ANOVA followed by the Sidak post hoc test for multiple comparison correction.

Different trends over time between groups (interaction) were assessed by two-way repeated measures ANOVA. Differences over time within each group were assessed by paired samples *t*-tests. One-way ANOVA of the percentual changes in the monocyte subsets was used to determine the magnitude of the exercise-induced effects and was followed by the Sidak post hoc test. Pearson correlation coefficients were used where appropriate. Multiple linear regression analysis was applied to investigate the independent association between exercise parameters and monocyte numbers. All analyses were performed using SPSS version 22 (SPSS Inc., Chicago, IL, USA) and a *P* value of <0.05 was considered statistically significant.

## 3. Results and Discussion

### 3.1. Results

#### 3.1.1. Baseline Characteristics of Subjects

Table 1 summarizes the baseline characteristics of the 3 groups. Age, sex, and BMI were comparable between groups. CKD patients presented with a mean eGFR 44.4 ± 19.7 mL/min/1.73 m^2^. CHF patients, with a mean LVEF of 31.1 ± 10.6%, were characterized by elevated atrial and left ventricular filling pressures.

All subjects performed a maximal exercise test, as was objectified by a RER value > 1.15. Aerobic exercise capacity (VO_2_peak) and maximal workload were significantly lower in CKD and CHF patients compared to HS. Compared to CKD and HS, patients with CHF had a reduced hemodynamic response with a lower peak heart rate, systolic blood pressure, and circulatory power (Table 1).

#### 3.1.2. Distribution of Monocyte Subsets and Levels of Inflammatory Proteins at Baseline

Total leukocyte count was within normal range in the 3 groups (4.3–10.0 10^6^/mL), but CHF patients had significantly higher white blood cell counts compared to CKD patients (Table 2). Whereas the distribution of neutrophils and lymphocytes was comparable between groups, CHF patients had a higher percentage of total monocytes and consequently a higher absolute count of all monocyte subsets. Distribution of the monocyte subsets was comparable between the groups: Mon1 comprised the largest percentage, followed by Mon3 and then Mon2 (Table 2).

Levels of MCP-1 were significantly higher in CKD and CHF compared to HS. IL-6 levels did not differ significantly between groups but were related to MCP-1 levels (*r* = 0.407, *P* < 0.001) and %Mon2 (*r* = 0.312, *P* = 0.031). Neither total leukocyte nor monocyte count was correlated to levels of IL-6 or MCP-1. Considering all groups, baseline levels of MCP-1 and IL-6 were significantly related to VO_2_peak (MCP-1  *r* = −0.330, *P* = 0.017; IL-6 *r* = −0.364, *P* = 0.013, resp.). No relation was found between leukocyte and monocyte counts or monocyte distribution with VO_2_peak.

#### 3.1.3. Effect of an Acute Exercise Bout on Leukocyte and Monocyte Distribution

Table 3 demonstrates that absolute monocyte count increased significantly and comparably in HS, CKD, and CHF patients 10 minutes following peak exercise. This was due to a general exercise-induced leukocytosis. Only in CHF, a significant increase in percentage of monocytes contributed to this leukocytosis.

**Figure 2:**
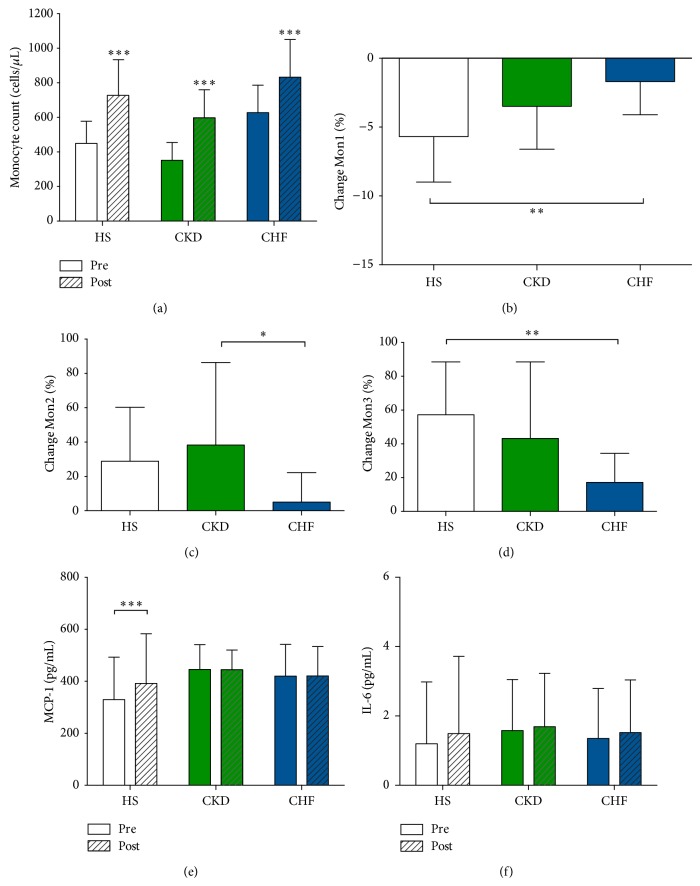
Magnitude of the exercise-induced effect on monocyte count, monocyte subsets, and serum MCP-1 and IL-6 levels. (a) Following peak exercise, absolute monocyte count increased significantly in all groups. (b–d) Within the total monocyte count, the percentage of Mon1 decreased in all three groups with a parallel increase in Mon2 and Mon3 (with exception of Mon2 in CHF). Between-group analysis revealed that the overall response of the monocyte subsets was comparable between HS and CKD but was significantly blunted for patients with CHF (*P* for interaction <0.05 for all subsets). (e) Following peak exercise, MCP-1 levels increased significantly in HS but remained unchanged in patients with CKD and CHF (*P* = 0.004 for interaction). (f) Increase in IL-6 levels were observed in all groups but failed to reach the level of significance (HS *P* = 0.08; CKD *P* = 0.644; CHF *P* = 0.063). Changes in monocyte subset are expressed as % change from baseline. ^***^ *P* < 0.001, ^**^ *P* < 0.01, and ^*^ *P* < 0.05.

In all 3 groups, percentage of Mon1 decreased, whereas Mon2 and Mon3 increased after a single bout (with exception of Mon2 in CHF, Table 3).  Figure 2 illustrates that the magnitude of this exercise-induced effect on monocyte subsets is different in the 3 groups. The overall exercise-induced response on monocyte subsets was comparable between HS and CKD but was blunted in patients with CHF (*P* for interaction <0.05 for all subsets). In CHF, the decrease in Mon1 was less prominent, the increase in Mon2 was nearly absent, and the increase in Mon3 again was less pronounced.

#### 3.1.4. Effect of an Acute Exercise Bout on Levels of MCP-1 and IL-6

Whereas an acute exercise bout did not alter levels of MCP-1 in patients with CKD and CHF, MCP-1 was significantly higher in HS following exercise (*P* = 0.004 for interaction, Figure 2(e)). Postexercise levels in MCP-1 were comparable between groups (*P* > 0.05). Increases in levels of IL-6 were observed in all groups but failed to reach the level of significance (HS *P* = 0.08; CKD *P* = 0.644; CHF *P* = 0.063, Figure 2(f)).

#### 3.1.5. Relation of Changes in Leukocytes and Monocyte Subsets with Baseline, Exercise, and Inflammation-Related Parameters

Considering all groups, baseline hemodynamic parameters such as systolic blood pressure, diastolic and systolic function, were strongly associated with the change of leukocyte and monocyte count and monocyte subset distribution after an acute exercise bout (Table 4). Subjects with an impaired diastolic function (higher E/é) and an impaired systolic function (lower LVEF) showed overall a less prominent change in leukocytes, monocytes, and monocyte subsets (decrease in %Mon1, increase in %Mon2 and %Mon3). Renal function (eGFR) or inflammatory status (MCP-1) did not relate to the magnitude of this effect.

Exercise-related hemodynamic parameters, such as VO_2_peak, circulatory power, and peak systolic blood pressure, were all related to changes in leukocyte or monocyte (subset) distribution (Table 4). To investigate the independent association between exercise-related parameters and monocyte subset change, a multiple linear regression analysis was performed, adjusting for age, sex, the use of beta-blockade, baseline systolic blood pressure, and diastolic and systolic function. VO_2_peak remained a strong predictor of the change in Mon1 (*β* = −0.495, *P* < 0.001) and Mon3 (*β* = 0.468, *P* = 0.001). In line, circulatory power remained negatively associated with the change in Mon1 (*β* = −0.461, *P* < 0.001) and positively with the change in Mon3 (*β* = 0.449, *P* < 0.001). For the change in Mon2, baseline LVEF emerged as the strongest response predictor (*β* = 0.441, *P* < 0.001). The same was true when correcting (in separate models) for the use of statins, ACE-inhibitors/ARB, diuretics, and acetylsalicylic acid.

A significant relation between the exercise-induced change in MCP-1 and Mon1 and Mon3 was observed (Table 4). However, after correction for VO_2_peak, which was significantly correlated to change in MCP-1 (*r* = 0.511, *P* < 0.001), this relation was lost.

### 3.2. Discussion

The present study investigates whether monocyte subset distribution and their response to an acute exercise bout are influenced by the presence of chronic low-grade inflammation (CKD and CHF), the presence of CKD* per se* (specific internal milieu), or CHF (specific hemodynamic alterations).

Several findings emerge from this study.

(i) The relative distribution of the monocyte subsets (Mon 1-2-3) is comparable in healthy subjects, CKD patients, and CHF patients. However, in CHF, the absolute monocyte count is significantly higher.
(ii) Following a single bout of maximal exercise, the percentage of Mon2 and Mon3 increases at the expense of a decrease in Mon1. This response is clearly blunted in patients with CHF despite the fact that they performed a maximal exercise test and that they reached a similar VO_2_peak and maximal workload compared to the CKD patients.
(iii) VO_2_peak and circulatory power emerge as strong predictors of the changes in Mon1 and Mon3, independent of beta-blocker use.

#### 3.2.1. Heterogeneity of Monocyte Subsets in Two Models of Chronic Disease with Low-Grade Inflammation

Before the nomenclature consensus in 2010, CD16^+^ monocytes (Mon2 and Mon3) were frequently studied collectively as proinflammatory monocytes, based on their cytokine expression profile and higher potency in antigen presentation. In addition to the fact that CD16^+^ monocyte count is increased in several inflammatory conditions, CD16^+^ monocytes have been clinically and mechanistically implicated in the pathophysiology of human cardiovascular disease [19].

The recognition of intermediate monocytes (Mon2) as a distinct subset urged for further unravelling of the function and behaviour of the different monocyte subtypes. Mon2 phenotypically resemble the previously reported proangiogenic monocytes, with the expression of receptors to proangiogenic factors (Tie2, CXCR4, and VEGFR1/2) [3, 20]. Indeed, bone marrow-derived CD14^+^Tie2^+^CD34^−^ cells are able to adhere on injured endothelium in a MCP-1-dependent manner, leading to reendothelialisation [21]. In contrast to their proangiogenic features, Mon2 possess a higher proinflammatory capacity compared to Mon3 [1] and selectively express CCR5, a marker that has been implicated in atherosclerosis [22]. These two characteristics could add to their possible unfavorable effect in cardiovascular disease. Indeed, in patients with CKD, in whom cardiovascular risk is known to be very high, as well as in patients at risk for coronary artery disease, elevated Mon2 counts are independent predictors of future cardiac events [23, 24]. In contrast, in the context of acute heart failure, lower Mon2 counts are independent predictors of increased mortality and repeat hospitalization [25]. These apparently conflicting data suggest diverse roles of the Mon2 subsets in different underlying disease processes.

In the present study, we show that the distribution of the 3 monocyte subsets (Mon1 > Mon3 > Mon2) is maintained in the presence of CKD or CHF. For CHF, this confirms the findings of Wrigley et al. [25], but it is in contrast with previous reports on a higher percentage of CD14^++^CD16^+^ monocytes [26] or the CD14^dim⁡^CD16^+^ subset [27] in patients with CHF in comparison to healthy subjects. These discrepant findings possibly could be explained by the use of a different immunophenotypical definition between studies or by differences in the studied populations (older patients and a predominance of ischemic cardiomyopathy in the study by Barisione et al.). In analogy to the study of Wrigley et al., we used a multiparametric flow cytometric technique with an iterative gating strategy for enumeration of the different subsets, whereas the other studies used a straightforward two-colour panel approach. As illustrated by Zawada et al. [28], a pan-monocytic marker (CD86) is required to correctly identify the monocytes and to distinguish the CD16^+^ monocytes from other CD16 expressing leukocytes, such as neutrophils and natural killer cells. Second, we applied a colouring and gating strategy based on the differential expression of CCR2 for correct distinction between Mon2 and Mon3, adapted from the publication by Shantsila et al. [3].

In patients with CHF total monocyte count was increased, resulting in a parallel increase of all 3 subsets counts, again confirming the data of Wrigley et al. [25]. Limited data in CHF suggest that an increase in total monocyte number predicts worse outcome [29]. Such an association is far less explored in CKD and healthy subjects [30]. It is plausible that, in our study, we underestimated monocyte count in CKD patients, since we preselected CKD patients with the best cardiovascular prognosis by excluding any cardiovascular history. Therefore, total monocyte counts were rather low and no increase in Mon2 count was detected in CKD.

#### 3.2.2. The Effect of an Acute Exercise Bout on Monocyte Subsets is Attenuated in CHF

It has been shown previously in healthy subjects that monocyte subsets behave differently in response to a physical stressor. Following exercise, CD16^+^ monocytes are preferentially mobilized from the marginal pool where they are sequestered because of a high expression of adhesion molecules like very late antigen-4 and CD11d [31–34]. These studies were performed before the nomenclature consensus in 2010 and only refer to 2 monocyte subpopulations (CD14^+^CD16^+^ and CD14^+^CD16^−^) without further subdivision of the CD16^+^ cells. Later studies, allowing for the trichotomy of monocytes, offered more insight in Mon2 and Mon3 behaviour following exercise of moderate intensity [35] or a maximal exercise bout [16] in healthy volunteers.

Up to now, no data existed on the acute exercise-induced monocyte trafficking in CKD or CHF, both conditions that benefit from exercise training programs in terms of lowering cardiovascular risk [9, 36]. In the present study, a similar increase of Mon2 and Mon3 was confirmed in CKD and in HS. However, in CHF, the overall exercise-induced response of monocyte subsets was clearly blunted despite comparable exercise-related parameters as the CKD patients.

To investigate whether this could be related to a different chemotactic response, serum levels of IL-6 and MCP-1 were quantified before and immediately after peak exercise. *MCP*-1, a CCR2 ligand, plays an important role in monocyte mobilization and their selective recruitment into tissues [37]. Besides production by inflammatory cells and endothelial cells, MCP-1 is a contraction-regulated myokine with a possible role in the exercise-induced changes in the immune system [38]. Following exercise at moderate intensity, mRNA expression of MCP-1 is upregulated in skeletal muscle cells, coinciding with an increased serum concentration that is even more pronounced after high intensity exercise [39]. In this study, a short bout of strenuous exercise elicits a significant increase in MCP-1 in healthy subjects, whereas no change was observed in CKD or CHF patients. Hypothetically, this could be attributed to the higher observed levels at baseline, to a reduced vascular shear stress, to the short exercise duration (8–10 min), or to a decreased muscle mass.* Interleukin*-*6* is another contraction-regulated myokine. The release of  IL-6 from contracting skeletal muscle [14] may facilitate a broad anti-inflammatory response via effects on liver as well as on different leukocyte populations (reviewed in [40]). The magnitude of this effect is affected by the mode, intensity, and duration of exercise [41]. In this study, we detected only a small, nonsignificant increase in IL-6 following exercise, possibly because of the short duration of exercise. Nevertheless, the increase in IL-6 significantly correlated with the magnitude of total leukocyte increase. Taken together, a differentially regulated chemotactic response of MCP-1 or IL-6 is not the only explanation for the observed between-group variations in monocyte subsets following strenuous exercise.

Another explanation is that the blunted hemodynamic response during exercise in CHF (lower cardiac output, lower peak heart rate, and peak systolic blood pressure) is insufficient to recruit Mon2 and Mon3 that are avidly adhered to activated vascular endothelium. The observed strong association between peak heart rate, circulatory power, and VO_2_peak supports this hypothesis. Release of CD16^+^ monocytes into the circulation is known to be in part catecholamine dependent [31], but the strong relationship between circulatory power and VO_2_peak and the response in monocyte subsets appeared to be independent of beta-blocker use. In conclusion, the release of Mon2 and Mon3 in the circulation, at the expense of the percentage Mon1, is strongly driven by hemodynamic responses to exercise, which could explain the blunted response observed in CHF.

#### 3.2.3. Limitations

In the present study, the effects of strenuous exercise were assessed 10 minutes after peak exercise. However, it is known that the exercise-induced effect on leukocytes follows a biphasic pattern, characterized by an immediate and delayed response (2–4 hours after exercise). In future studies, it would be interesting to investigate the time course of monocyte subsets over a longer time period.

## 4. Conclusion

Monocytes play a substantial role in systemic low-grade inflammation that is associated with cardiovascular disease, with distinct functions for the 3 monocyte subsets. Whereas the anti-inflammatory effect of exercise training is well established in chronic diseases such as CKD and CHF, the response to acute exhaustive exercise is far less explored.

This study is the first to show that CKD patients, despite a lower exercise capacity and presence of low-grade inflammation, show a comparable acute exercise-induced change in monocyte subsets as healthy subjects. This effect is characterized by an increase in proangiogenic and proinflammatory Mon2 and Mon3, at the expense of Mon1. However, in CHF patients this effect is clearly attenuated and is strongly driven by a decreased hemodynamic response to exercise. Our findings offer more insight into the dynamic inflammatory response of acute exercise in different disease states, which is essential for the further unravelling of the mechanisms underlying the long-term beneficial effects of exercise training.

## Figures and Tables

**Table 1:** Baseline characteristics of subjects.

	HS (*n* = 15)	CKD (*n* = 20)	CHF (*n* = 20)	*P* value
Age (years)	43.5 ± 5.0	51.3 ± 15.6	51.2 ± 9.3	0.08
Gender (F/M)	6/9	12/8	7/13	0.25
Body mass index (kg/m^2^)	24.2 ± 2.3	26.1 ± 5.1	26.6 ± 3.8	0.18
Systolic BP (mmHg)	123 ± 13	122 + 13	102 ± 18^∗†^	<0.001
Diastolic BP (mmHg)	77 ± 9	77 ± 9	69 ± 10^∗†^	<0.05
Biochemistry				
eGFR (mL/min/1.73 m^2^)	99.0 ± 11.3	44.4 ± 19.7^∗^	88.7 ± 12.9^†^	<0.001
Total cholesterol (mg/dL)	172.7 ± 22.2	173.8 ± 24.1	178.3 ± 8.9	0.83
HDL (mg/dL)	58.9 ± 13.6	57.7 ± 18.8	46.8 ± 11.3^∗†^	<0.05
LDL (mg/dL)	111.4 ± 34.8	98.4 ± 20.7	99.3 ± 24.7	0.32
Echocardiography				
LVEF (%)	65 ± 0	62.9 ± 8.2	31.1 ± 10.6^∗†^	<0.001
LVEDD (mm)	49.8 ± 4.4	47.8 ± 4.9	59.9 ± 13.2^∗†^	<0.001
E/$\overset{´}{\text{e}}$	9.2 ± 1.3	9.7 ± 2.4	17.4 ± 8.9^∗†^	<0.001
RAP (mmHg)	5.4 ± 1.4	4.6 ± 1.1	6.5 ± 2.9^†^	<0.001
Medication use				
Beta-blockers (%)	/	35	100	<0.001
Diuretics (%)	/	15	70	<0.05
Acetylsalicylic acid (%)	/	5	40	<0.05
Statins (%)	/	55	50	0.5
ACE-inhibitors/ARB (%)	/	60	95	<0.05
CPET-derived parameters				
RER	1.24 ± 0.11	1.38 ± 0.13^∗^	1.34 ± 0.12	<0.05
VO_2_peak (mL/kg/min)	37.40 ± 9.38	25.54 ± 7.54^∗^	22.19 ± 5.96^∗^	<0.001
% predicted VO_2_peak (%)	107 ± 22	84 ± 20^∗^	71 ± 13^∗^	<0.001
Maximal workload (Watt)	246 ± 85	152 ± 50^∗^	132 ± 49^∗^	<0.001
% predicted Wattmax (%)	123 ± 26	95 ± 29^∗^	81 ± 19^∗^	<0.001
VO_2_ at AT (mL/kg/min)	31.42 ± 8.14	24.96 ± 6.75	20.08 ± 6.36^∗^	0.001
Peak heart rate (bpm)	170 ± 14	154 ± 27	135 ± 21^∗†^	<0.001
Peak systolic BP (mmHg)	189 ± 35	188 ± 21	137 ± 27^∗†^	<0.001
Peak diastolic BP (mmHg)	80 ± 11	82 ± 16	73 ± 14	0.156
Work economy (Watt/(mL/kg/min))	6.8 ± 1.5	6.1 ± 1.3	5.9 ± 1.3	0.19
Circulatory power (mmHg*·*mLVO_2_/(kg/min))	7017 ± 2504	4828 ± 1669^∗^	3097 ± 1191^∗†^	<0.001
*T*_1/2_ VO_2_peak (seconds)	120 ± 45	176 ± 43^∗^	199 ± 32^∗^	<0.001
Exercise duration (sec)	749 ± 230	424 ± 130^∗^	447 ± 120^∗^	<0.001

Data are mean ± SD. *P* value for comparison of groups (ANOVA). ^∗^Different from HS, *P* < 0.05. ^†^Different from CKD, *P* < 0.05.

BP: blood pressure; eGFR: estimated glomerular filtration ratio; HDL: high-density lipoprotein; LDL: low-density lipoprotein; LVEF: left ventricular ejection fraction; LVEDD: left ventricular end-diastolic diameter; RAP: right atrial pressure; RER: respiratory exchange ratio; VO_2_peak: peak oxygen uptake; VO_2_ at AT: oxygen uptake at anaerobic threshold; *T*_1/2_ VO_2_peak: VO_2_peak half-time; ACE: angiotensin converting enzyme; ARB: angiotensin receptor blocker.

**Table 2:** Distribution of monocyte subsets and levels of inflammatory proteins at baseline.

	HS (*n* = 15)	CKD (*n* = 20)	CHF (*n* = 20)	*P* value
*Leukocytes *				
WBC count (10*E*6/mL)	7.17 ± 1.60	5.76 ± 1.45	8.24 ± 1.82^†^	<0.001
*WBC formula (% of leukocytes) *				
%neutrophils	65.1 ± 10.6	63.1 ± 7.8	59.8 ± 9.7	0.24
%lymphocytes	24.8 ± 8.7	24.2 ± 6.8	27.5 ± 8.3	0.38
%monocytes	6.28 ± 1.24	6.16 ± 1.35	7.72 ± 1.88^∗†^	<0.05
*Monocytes *				
Monocyte count (cells/*μ*L)	450 ± 128	352 ± 103	628 ± 159^∗†^	<0.001
*Monocyte subsets (% of monocytes) *				
%Mon1	88.09 ± 4.73	88.48 ± 4.27	87.34 ± 3.54	0.67
%Mon2	4.51 ± 2.05	3.56 ± 1.69	4.74 ± 2.46	0.18
%Mon3	7.39 ± 3.17	7.95 ± 3.61	7.92 ± 2.19	0.83
*Monocyte subsets (cells/L) *				
Mon1	395.2 ± 107	311.6 ± 93.8	550.3 ± 143.9^∗†^	<0.001
Mon2	20.7 ± 13.5	13.1 ± 8.7	29.3 ± 17.1^†^	<0.01
Mon3	34.1 ± 20.9	27.3 ± 13.9	49.3 ± 17.3^∗†^	<0.01
*Inflammatory cytokines *				
MCP-1 (pg/mL)	330 ± 163	446 ± 95^∗^	420 ± 119^∗^	0.028
IL-6 (pg/mL)	1.17 ± 1.72	1.63 ± 1.43	1.41 ± 1.33	0.69

Data are mean ± SD. *P* value for comparison of groups (ANOVA). ^∗^Different from HS, *P* < 0.05. ^†^Different from CKD, *P* < 0.05.

MCP-1: monocyte chemoattractant protein-1; IL-6: interleukin-6.

**Table 3:** Effect of an acute exercise bout on leukocyte and monocyte distribution.

	Pre	Post	% change	*P* value for pre-postcomparison	*P* value for interaction
*Leukocytes (10E6/mL) *					
HS	7.17 ± 1.60	11.79 ± 1.99	67.28 ± 26.54	<0.001	<0.001
CKD	5.76 ± 1.45	9.95 ± 2.39	76.65 ± 37.1	0.006	
CHF	8.24 ± 1.82	10.21 ± 1.66	26.47 ± 18.0^∗†^	<0.001	
*Monocytes (cells/*μ*L) *					
HS	450 ± 128	728 ± 206	62.79 ± 21.36	<0.001	0.26
CKD	352 ± 103	598 ± 162	75.95 ± 44.68	<0.001	
CHF	628 ± 159	833 ± 218	34.85 ± 23.89	<0.001	
*Monocytes (% of leukocytes) *					
HS	6.28 ± 1.24	6.13 ± 1.16	−1.92 ± 9.04	NS	<0.001
CKD	6.16 ± 1.35	6.17 ± 1.93	0.09 ± 17.89	NS	
CHF	7.72 ± 1.88	8.21 ± 2.15	6.55 ± 9.74	0.024	
*Mon1 (% of total monocytes) *					
HS	88.09 ± 4.73	83.12 ± 5.98	−5.7 ± 3.3	<0.001	0.001
CKD	88.48 ± 4.27	85.27 ± 3.73	−3.5 ± 3.1	<0.001	
CHF	87.34 ± 3.54	85.86 ± 4.25	−1.7 ± 2.4^∗^	0.004	
*Mon2 (% of total monocytes) *					
HS	4.51 ± 2.05	5.62 ± 2.30	28.9 ± 31.4	<0.001	0.002
CKD	3.56 ± 1.69	4.43 ± 1.62	38.3 ± 48.1	0.001	
CHF	4.74 ± 2.46	4.77 ± 2.22	5 ± 17.2^†^	NS	
*Mon3 (% of total monocytes) *					
HS	7.39 ± 3.17	11.26 ± 4.59	57.2 ± 31.3	<0.001	0.004
CKD	7.95 ± 3.61	10.29 ± 3.0	43.2 ± 45.3	<0.001	
CHF	7.92 ± 2.19	9.37 ± 3.13	17.2 ± 17.2^∗^	<0.001	

Data are mean ± SD. *P* value for within-group (paired samples *t*-test) and between-group comparison (repeated measures ANOVA). ^∗^Different from HS, *P* < 0.05. ^†^Different from CKD, *P* < 0.05.

**Table 4:** Relation of changes in monocyte subsets with baseline, exercise, and inflammation-related parameters.

	Change WBC count		Change Mon count		Change %Mon1		Change %Mon2		Change %Mon3	
	*r*	*P*	*r*	*P*	*r*	*P*	*r*	*P*	*r*	*P*
*Baseline parameters *										
Systolic BP	**0.438**	<0.001	**0.366**	0.007	−0.215	0.122	0.213	0.125	**0.364**	0.007
Diastolic dysfunction (E/$\overset{´}{\text{e}}$)	**−0.403**	0.003	**−0.333**	0.015	**0.367**	0.007	**−0.327**	0.017	**−0.359**	0.008
Systolic function (LVEF)	**0.579**	<0.001	**0.429**	<0.001	**−0.422**	0.002	**0.430**	<0.001	**0.378**	0.005
eGFR	**−0.28**	0.038	−0.245	0.072	−0.111	0.420	−0.177	0.195	0.075	0.588
MCP-1	−0.077	0.582	−0.066	0.633	0.075	0.589	0.161	0.245	−0.050	0.722
*Exercise-related hemodynamic parameters *										
VO_2_peak	**0.418**	0.002	**0.308**	0.025	**−0.536**	<0.001	0.218	0.117	**0.504**	<0.001
Circulatory power	**0.477**	<0.001	**0.309**	0.031	**−0.466**	<0.001	0.229	0.114	**0.451**	<0.001
Peak HR	**0.588**	<0.001	**0.418**	0.002	**−0.409**	0.002	0.223	0.101	**0.311**	0.021
Peak systolic BP	**0.523**	<0.001	**0.323**	0.021	**−0.389**	0.005	**0.291**	0.038	**0.352**	0.011
*Inflammation-related parameters *										
IL-6 change	**0.323**	0.037	0.046	0.774	−0.093	0.557	0.015	0.923	−0.009	0.954
MCP-1 change	0.205	0.140	0.190	0.172	**−0.391**	0.004	−0.027	0.846	**0.319**	0.020

BP: blood pressure; LVEF: left ventricular ejection fraction; VO_2_peak: peak oxygen uptake; HR: heart rate; IL-6: interleukin-6; MCP-1: monocyte chemoattractant protein-1.

*r*: Pearson correlation coefficient. Significant correlations are presented in bold.
